# Pangenome analysis reveals both niche-specific “specialists” and microbial “side hustlers” in colorectal cancer microbiomes

**DOI:** 10.1080/19490976.2026.2728331

**Published:** 2026-09-15

**Authors:** Justin Lee, Samuel Minot, Neelendu Dey

**Affiliations:** a Creighton School of Medicine, Phoenix, AZ, USA; b Cirro Bio, Inc., Seattle, WA, USA; c Microbiome Research Initiative, Fred Hutchinson Cancer Center, Seattle, WA, USA; d Translational Science and Therapeutics Division, Fred Hutchinson Cancer Center, Seattle, WA, USA; e Department of Medicine, Division of Gastroenterology, University of Washington, Seattle, WA, USA

**Keywords:** Colorectal cancer, gut microbiome, pangenomes, metagenomics, cancer prevention, microbial biomarker

## Abstract

The gut microbiome is reproducibly implicated in colorectal cancer (CRC), yet the inter-study and interpersonal variability of certain species associations suggests that CRC microbiomes may be defined by convergent functional states achievable by phylogenetically diverse organisms. Distinguishing lineage-conserved “specialists” from taxonomically diverse “side hustlers”—organisms whose shared functional traits are dispersed across phylogenetically distant lineages—offers complementary translational insights: “specialists” are primary candidates for lineage-targeted biomarkers and inhibitors, while the shared functional architecture of “side hustlers” may reveal high-priority potential therapeutic targets robust to inter-individual variability. Here, we quantify phylogenetic coherence (monophyly) of 3,711 co-associated gene bins (CAGs) across 13 bacterial species and evaluate CRC associations across three independent cohorts, identifying hundreds of CAGs associated with CRC or health across a spectrum of monophyly scores, indicating that both states harbor a mixture of “specialist” and “side hustler” gene content. Strikingly, in *Faecalibacterium prausnitzii*—a species with a complex relationship to CRC—health-associated CAGs exhibited significantly higher monophyly scores than CRC-associated CAGs, consistent with health-linked traits being lineage-conserved while CRC-linked traits behave as polyphyletically distributed, potentially mobile “side hustlers.” Across multiple CRC-associated species, we observe functional convergence in gene bins encoding Type IV secretion systems (T4SS), TonB-dependent receptors, and RagB/SusD nutrient uptake proteins. *Fusobacterium animalis* strains encode T4SS elements across bins with variable phylogenetic origins, representing simultaneous “specialist” and “side hustler” strategies within a single species. Pairwise interaction analysis further reveals synergistic interspecies associations, including co-occurrence of *F. animalis* and *Clostridium scindens* gene bins associated with a CRC probability of > 90%, suggesting that microbial “side hustlers” may amplify oncogenic risk through ecological interactions invisible to species-level analysis. These findings provide proof-of-principle that an ecological and evolutionary lens on the CRC microbiome can identify shared functional vulnerabilities and lineage-specific targets for microbiome-based cancer prevention.

## Introduction

Mounting evidence implicates the gut microbiome as a key player in the pathogenesis of colorectal cancer (CRC),^1-3^ yet translating these associations into microbiome-based CRC prevention tools has been challenging. Although some specific bacterial taxa are reproducibly linked to CRC, many gut bacterial species and sub-species associations often differ by study, varying by geography, demographics, and clinical history.^4-6^ This variability may reflect a fundamental property of CRC microbiomes as ecosystems defined by a convergent functional state achievable by phylogenetically diverse taxa i.e. a *polyphyletic* community analogous to microbiomes in inflammatory bowel diseases.^7^ ^,^ ^8^

Historically, efforts to dissect the role of the microbiome in disease have relied primarily upon taxonomic profiling.^9^ While this approach has identified several reproducible CRC-associated organisms, it implicitly treats species abundance as a proxy for disease-relevant functional capacity. This assumption is limited in two important aspects. First, bacterial species exhibit substantial strain-level genomic variation, such that genes contributing to host interactions, nutrient acquisition, or microbial fitness may be present in only a subset of strains.^10^ Second, phylogenetically different organisms may encode similar functions through the influence of convergent evolution aided by horizontal gene transfer (HGT). Species-level profiling may therefore overlook both disease-associated traits restricted to particular strains and shared functional capacities spread across taxonomically diverse organisms.

These limitations motivate a functional evolutionary framework that evaluates disease-associated gene content independently of species abundance alone. Such a framework can determine whether an associated trait is conserved within a specific lineage or dispersed among phylogenetically distant strains, thereby distinguishing lineage-specific associations from strain-level differences and broader functional convergence. To apply this framework, we analyzed co-associated gene bins (CAGs) identified in our previous work.^3^ ^,^ ^5^ CAGs are clusters of protein-coding genes that co-occur and are quantified together, analogous to a species-level operational taxonomic unit but resolved to the sub-species gene content level. This approach can detect association carried by only a small subset of strains and can identify related functional elements that recur across different genomic backgrounds. We then measured the phylogenetic coherence of each CAG relative to the corresponding core-genome phylogeny to evaluate whether CRC- and health-associated gene content was predominantly lineage-conserved or phylogenetically dispersed.

A central hypothesis of our study is that the CRC microbiome is defined by convergent functional traits. In pursuing this investigation, we acknowledge that the alternative hypothesis—that the CRC microbiome is defined by specific CAGs—is not mutually exclusive and in fact supported by evidence including our own.^3^

Our investigation is motivated by two goals with complementary translational implications. First, we sought to identify the shared functional architecture of the CRC microbiome (“side hustlers”), reasoning that this is embedded in the polyphyletic functional gene content of CRC-associated CAGs, reflecting opportunistic adaptation conferring fitness.^11^ Second, by identifying monophyletic features of niche-specific CRC-associated CAGs (“specialists”), we aim to pinpoint lineage-specific traits that may serve as targets for novel microbiome-based CRC screening or therapeutic intervention. We envision a combination of “specialists” and “side hustlers” in CRC and in health, and we propose that distinguishing these two modes matters from a translational perspective: lineage-conserved “specialist” traits primarily offer reproducible targets for biomarker development (and, if associated lineage-specific metabolic traits can be identified, could also potentially motivate design of taxon-specific small molecule inhibitors), while the shared functional architecture of “side hustler” traits may reveal pharmacological intervention targets that are robust to the inter-study interspecific variability (and could also lend itself to biomarker design identifying a functional trait). By framing CRC microbiome research in terms of the evolutionary and ecological distinction between monophyletic and polyphyletic association, our work offers both a mechanistic proof-of-principle for linking gut microbial ecology to colorectal carcinogenesis and a principled strategy for translating microbiome research into clinical tools.

## Results

### A pan-genome analysis of gut bacteria associated with CRC, health, or both in three independent cohorts

**Figure 1.**
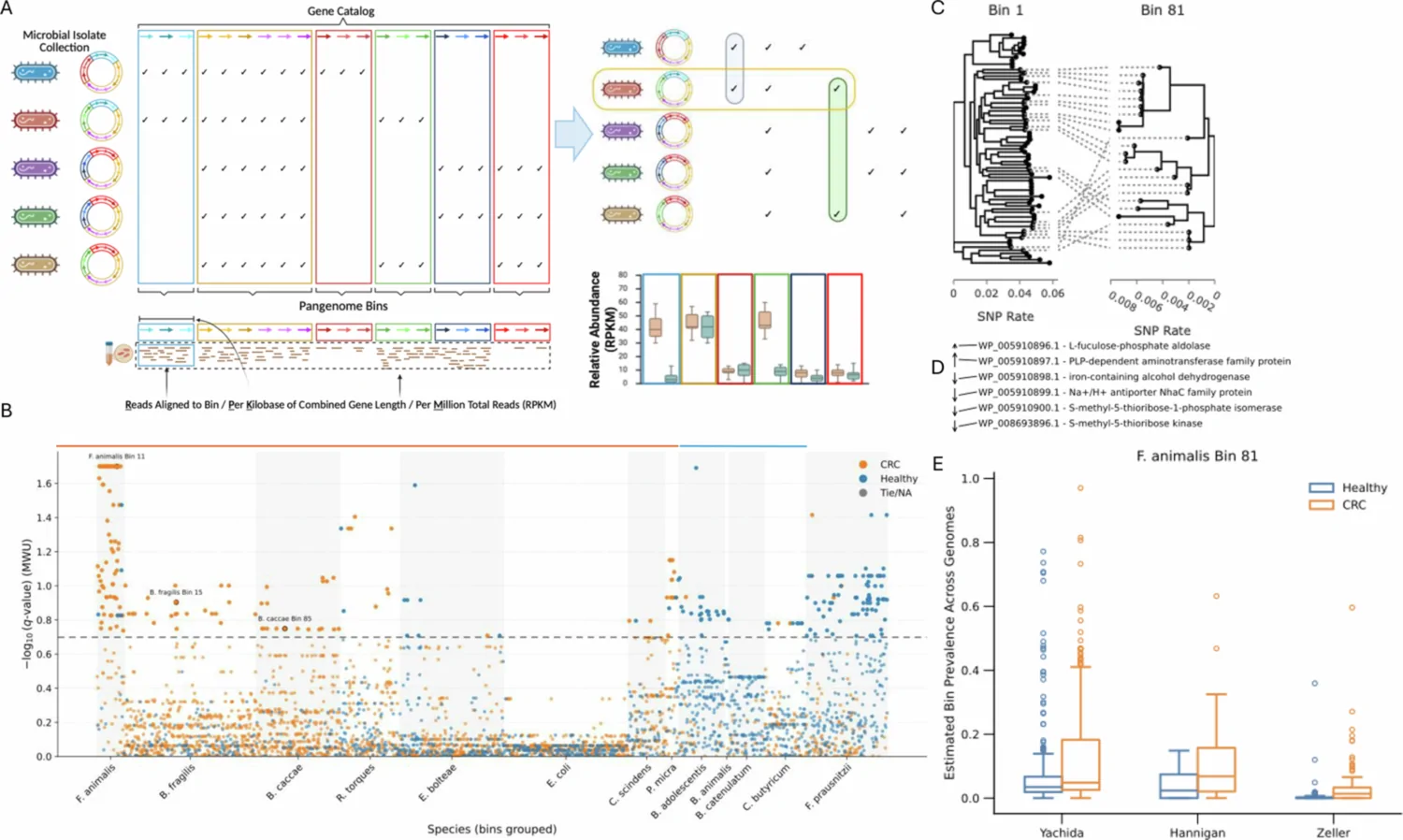
Pangenome gene bin abundance in CRC microbiome. **a**, Schematic of pangenome CAG construction and gene bin abundance calculations. **b**, Manhattan plot of all pangenome bins included in our species-based analysis. Each dot represents a gene bin whose association was calculated using the Mann-Whitney U test. The horizontal dotted line represents an FDR-corrected significance threshold of *q* ≤ 0.2. Species association (CRC/health) is denoted through the colored line at the plot’s upper border. **c,** Phylogenetic tree comparing *F. animalis* (bin 81) to a reference genome (bin 1). The degree of vertical separation between nodes is representative of genomic dissimilarity and dotted lines indicate shared genomes of origination. **d,** Annotated genes contained within *Fusobacterium* (bin 81). **e,** Genomic abundance of *Fusobacterium* (bin 81), stratified via condition (CRC/health) and experimental study.

In order to assess gene level contributions to CRC, we generated pangenome reference databases for a set of 12 bacterial taxa which had been previously identified as either enriched in CRC (*n* = 8) or in health (*n* = 4) (**Table S1**).^3^ ^,^ ^12^ ^,^ ^13^ We also studied *Faecalibacterium prausnitzii* (**Table S1**), which has been described in a complex relationship with CRC based on a meta-analysis of published microbiome studies.^3^ Each species’ pangenome was constructed as a catalog of non-redundant protein-coding genes clustered into bins based on co-abundance patterns across reference genomes (Figure 1A). Three published microbiome survey datasets comprising a total of 379 individuals with CRC and 347 healthy controls, drawn from three distinct geographic and dietary contexts (Japan, the United States, and Europe), were used to quantify gene bin abundances in CRC and in health (Yachida 2019;^14^ Hannigan 2018,^15^ Zeller 2014.^16^) The relative abundances of each gene bin were calculated independently for each microbiome sample as the number of reads aligned uniquely to genes within the bin, divided by the aggregate gene length, normalized to the number of reads aligned to the total pangenome (reads per kilobase per million reads [RPKM]) (Figure 1A).

In the Yachida dataset, 509 out of a total 3,711 (13.7%) gene bins differed significantly between CRC and controls (300 CRC-enriched, 209 health-enriched) (*p* < 0.05, Mann-Whitney U) across all 13 species (**Table S2A**). In the Hannigan dataset, 170 out of a total 3,509 (4.8%) gene bins differed significantly between CRC and controls (85 CRC-enriched, 85 health-enriched) (*p* < 0.05, Mann-Whitney U) across all 13 species (**Table S2A**). In the Zeller dataset, 885 out of a total 3683 (24.0%) gene bins differed significantly between CRC and controls (*p* < 0.05, Mann-Whitney U) across all 13 species (**Table S2A**). Overall, these observations mirrored our prior report in which we found that 2,319 out of 22,295 CAGs (10.4%) identified across 3 publicly available datasets were associated with either CRC or health.^3^

After the Benjamini-Hochberg procedure was applied to the Yachida dataset to correct for multiple hypothesis testing and minimize false discovery rate (FDR-BH threshold set to 0.20, Mann-Whitney U), 276 of a total 3,711 (7.4%) pangenome bins remained significantly different between CRC and controls (149 CRC-enriched, 127 health-enriched) across all 13 species (Figure 1B, **Table S2B)**. For the species that contained significantly associated bins, the number of significant bins ranged from 2 (*B. animalis*) to 83 (*F. animalis*), with 11 species having at least two significant bins and 10 having both CRC- and health-enriched bins, indicating substantial species-level heterogeneity.

Among the 3,505 gene bins quantified in all three cohorts, 92 gene bins were positively associated with CRC in exactly two cohorts, compared with a permutation-derived mean of 77.5, placing the observed count at the 99th percentile of the null distribution (**Table S2C)**. Two were positively associated with CRC in all three, compared with a permutation-derived mean of 1.16 (88th percentile of the null distribution).

We interpret this pattern in the context of substantial technical and biological heterogeneity among the three cohorts. Per-cohort detection rates differed several-fold (4.8% of gene bins significant in Hannigan versus 24.0% in Zeller), and the studies differ in sequencing platform, sequencing depth, and source population. Concordance at the level of individual gene bins is therefore not the expected outcome of adding cohorts, and the central claim of this work concerns convergence at the level of encoded function rather than reproducibility of specific gene bins across populations.

To illustrate the genomic contexts of these associations, we selected *F. animalis* bin 81, which was among the most strongly CRC-enriched gene bins, with consideration of all three studies (**Fig. S3, Table S2D**). The *F. animalis* genomes containing this bin were distributed paraphyletically across 24 of the 73 reference genomes used to build the pangenome (Figure 1C), meaning that they were not exclusive to any single subspecies-level clade. The genes contained in this bin are located adjacently within all reference genomes, suggesting coordinated transcription as part of an operon, with annotations both for sugar metabolism and transport (Figure 1D). We estimate what proportion of *F. animalis* genome copies contain this operon by normalizing to the core genome bin, and we observe that bin 81 was present at similar proportions in each of the CRC and healthy groups respectively between the three studies (Figure 1E). The higher relative fitness of *F. animalis* strains containing this operon (as manifested by their larger share of the population) could have a causal connection to or from CRC, either by increasing the likelihood of its development or by conferring a growth advantage in the environment of a CRC microbiome.^17^

### Distributions of monophyly scores are comparable in CRC and health

**Figure 2.**
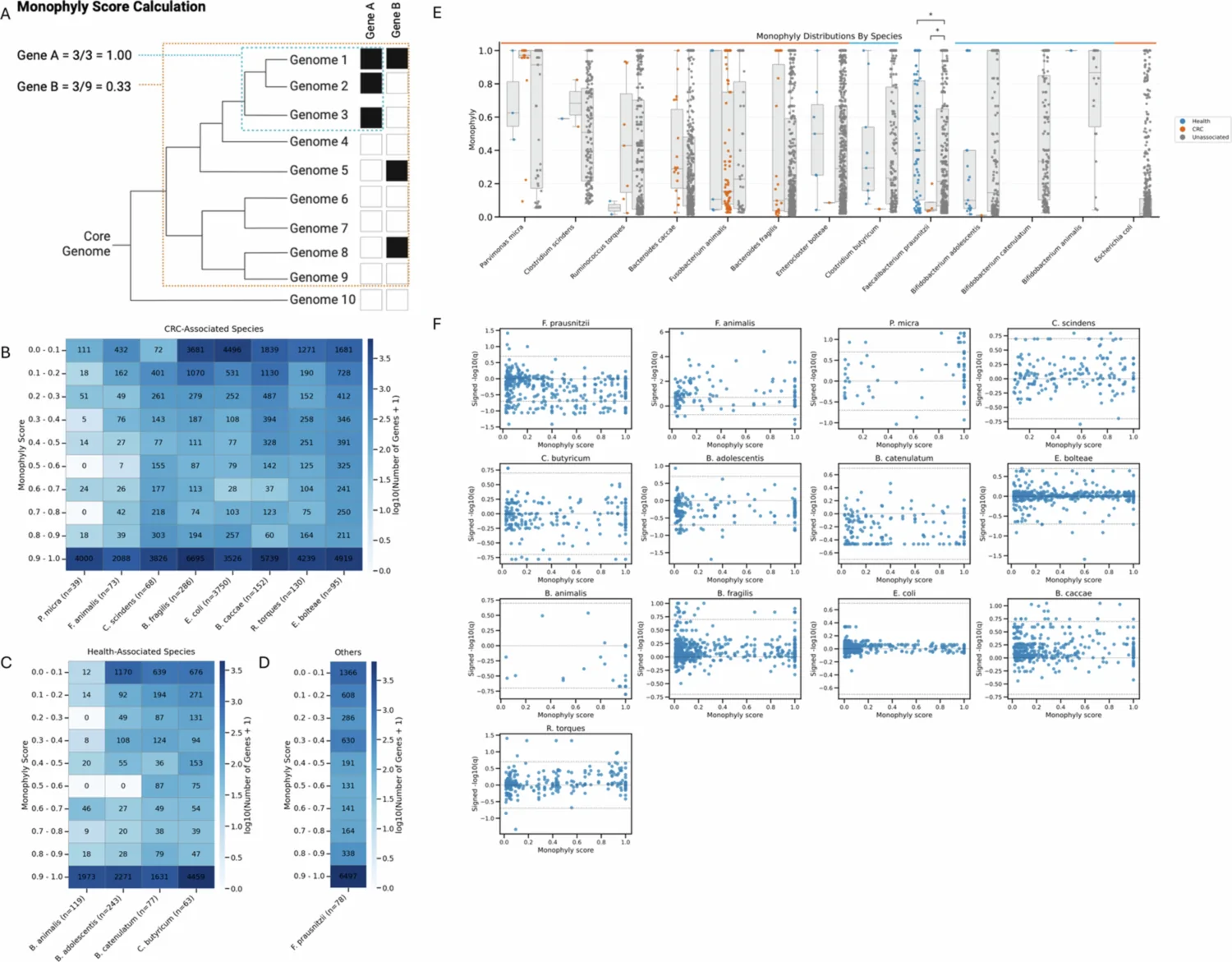
Monophyly patterns across CRC- and health-associated bacterial species. **a,** Diagram illustrating monophyly score calculations. **b,** Heatmaps of gene presence variation relative to monophyly for CRC-associated species, **c,** health-associated species, and **d,** *F. prausnitzii*. **e,** Pangenome bin monophyly distribution across all species. Bins passing FDR-corrected significance threshold of *q* ≤ 0.2 are colored according to their association. **f,** Relationship between monophyly score and signed -log_10_(*q*). Positive y values therefore indicate CRC association, whereas negative values represent health association. Horizontal dashed lines denote the significance threshold (*q* ≤ 0.2).

We next asked whether the CRC- and health-associated pangenome bins in general were representative of monophyletic clades (i.e., “specialists”) within each of the species included in this analysis. To assess the degree of monophyly observed for CRC- and health-associated pangenome bins, we defined a “monophyly score” that could be applied to any pangenome bin. This score was calculated as the proportion of genomes encoding the gene relative to the number of genomes present in the smallest monophyletic clade encompassing this gene. (Figure 2A). We interpret this score as a measure of phylogenetic coherence. A high score indicates that a gene bin is largely confined to a single lineage, consistent with vertical inheritance. A lower score indicates a broad distribution requiring repeated gene gain or loss across the species phylogeny. Although such a pattern is compatible with horizontal gene transfer, it may also arise from ancient polymorphism or differential gene loss and therefore does not establish HGT by itself. The distribution of genes by monophyly score varied widely across species, with similar degrees of variation observed in all species profiled (Figure 2B–D).

Health-associated bins of *F. prausnitzii* had higher overall monophyly scores compared to the unassociated bins (FDR-corrected *p* = 0.023, Mann-Whitney U), whereas CRC-associated bins of *F. prausnitzii* exhibited lower monophyly scores relative to unassociated bins (FDR-corrected *p* = 0.047; Figure 2E). These findings are consistent with health-associated features being found within lineage-conserved “specialist” clades, whereas the distribution of CRC-associated gene bins appears more consistent with polyphyletic, potentially mobile “side-hustling” genetic elements. This was a finding unique to *F. prausnitzii*, as the other species exhibited similar monophyly distributions regardless of CRC association. After correcting for multiple hypotheses, the monophyly scores for species other than *F. prausnitzii* were not significantly different between CRC and healthy microbiomes, or when compared to the unassociated bins (the null distribution for this test). Examining signed significance values as a function of monophyly score revealed a bimodal distribution, consistent with the coexistence of “specialist” and “side hustler” gene bins within each species, without clear enrichment of either pattern in CRC versus health (Figure 2E,F).

**Figure 3.**
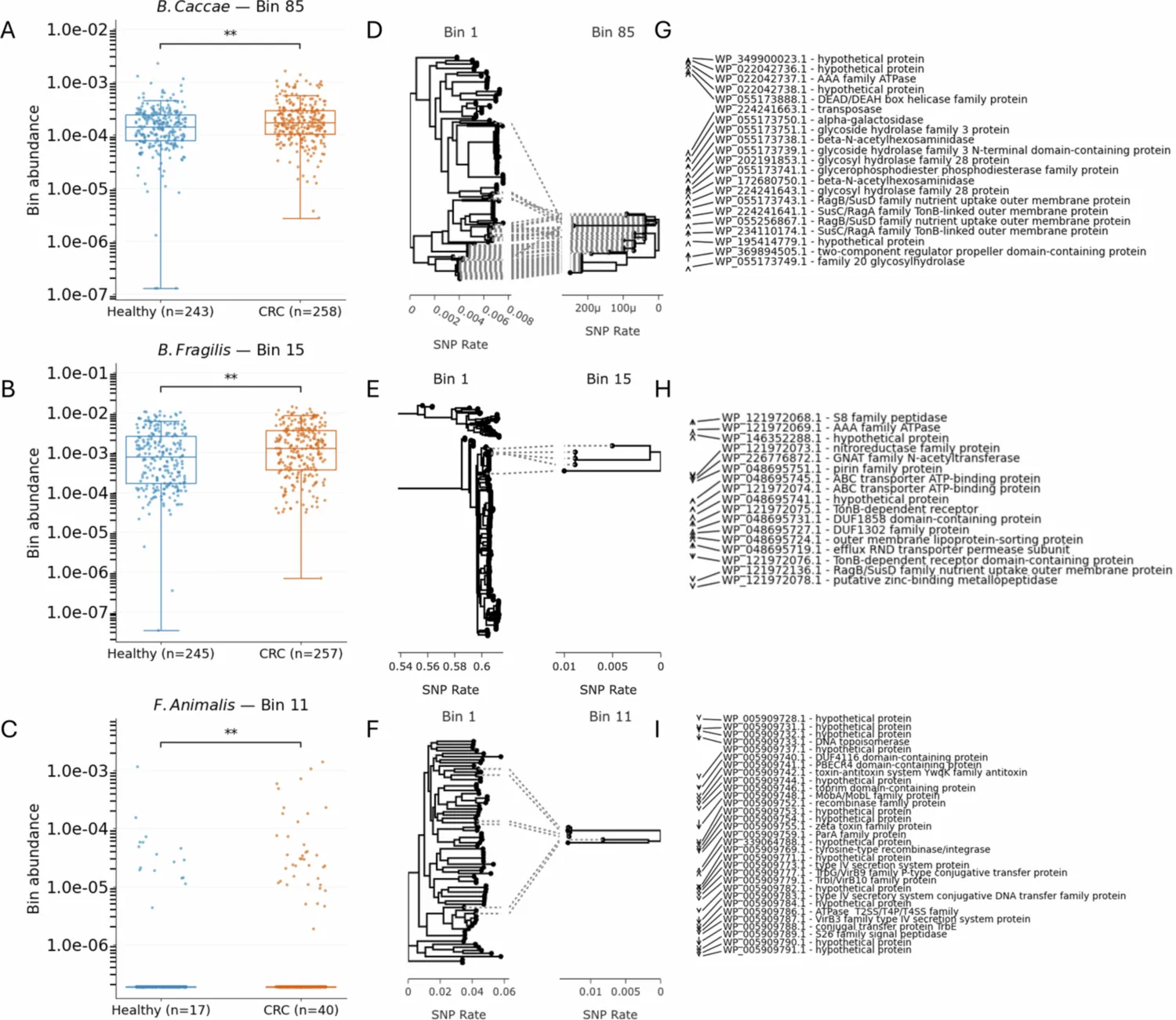
CRC-associated gene bins with low monophyly scores. **a-c**, Mann-Whitney U test-based significance comparison between bin abundances in healthy vs. CRC samples within the Yachida dataset. **d-f**, Phylogenetic comparisons between bins of interest and their reference genome. **g-i,** Annotated contents of the selected bins (3a-c).

Nonetheless, examination of individual CRC-associated gene bins revealed shared functional themes consistent with “side hustler” behavior, including nutrient acquisition and host-interaction machinery distributed across phylogenetically diverse species. Based on significance and functional annotation relevance, we highlight CRC-associated pangenome bins from *B. caccae* (bin 85, monophyly score = 0.27), *B. fragilis* (bin 15, monophyly score = 0.032), and *F. animalis* (bin 11, monophyly = 0.14), which were present at higher relative abundance in CRC samples compared to healthy controls (Figure 3). These gene bins contain genes annotated for RagB/SusD, TonB-dependent receptor, and Type IV secretion, respectively (Figure 3A–I). The paraphyletic distribution of these bins across each species is consistent with repeated gain or loss, potentially including horizontal acquisition, a theme that recurs across the CRC-associated pangenome and is examined further below.

### Fusobacterium pangenome gene bins encoding energy regulation and Type IV secretion are enriched in CRC

**Figure 4.**
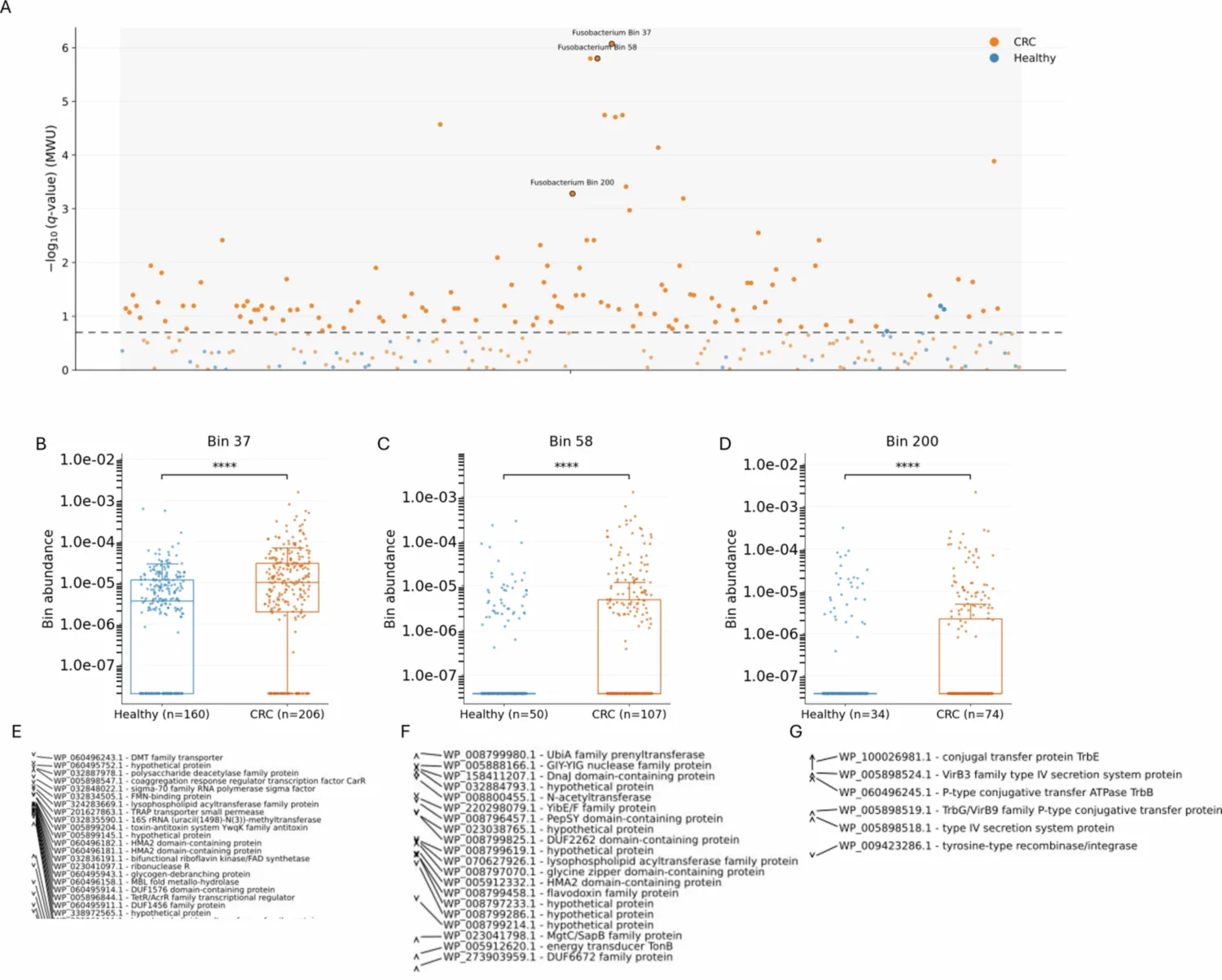
*Fusobacterium* bin and monophyly distribution. **a,** Manhattan plot of pangenome bins within the *Fusobacterium* genus. Each dot represents a gene bin whose association was calculated using Mann-Whitney U. The horizontal dotted line represents an FDR-corrected significance threshold of *q* ≤ 0.2. **b-d,** *Fusobacterium* abundance comparisons between healthy vs. CRC samples within the Yachida dataset (significance threshold *q* ≤ 0.2, calculated by Mann-Whitney U). **e-g**, Annotated contents of the selected bins shown in b-d.

In light of literature linking multiple species of *Fusobacterium* to GI cancers,^17^ ^,^ ^18^ we analyzed pangenome bins more broadly across the *Fusobacterium* genus. After multiple hypothesis testing correction (FDR-BH = 0.2), 121 bins were found to be CRC-associated, and 3 were health-associated (Figure 4A). The monophyly distribution of these bins showed no significant difference from the unassociated bins, suggesting that CRC-associated *Fusobacterium* gene content does not show genus-level enrichment for either “specialist” or “side hustler” behavior in aggregate (**Fig. S4**). From the group of gene bins enriched in CRC microbiomes, we selected for further evaluation three bins of high significance and functional annotation relevance (Figure 4B–D). These bins had monophyly scores ranging from 0.23 (bin 200) to 0.44 (bin 37) to 0.76 (bin 58), highlighting the diversity of phylogenetic distributions of CRC-associated genes even within a genus: some strongly CRC-associated bins are constrained to local regions within the *Fusobacterium* phylogenetic tree, whereas others are dispersed polyphyletically. Functionally relevant genes encoded in these gene bins included coaggregation response regulator (Figure 4E), energy transducer TonB (Figure 4F), and Type IV secretion system (Figure 4G), respectively (reviewed in *Discussion*). Type IV secretion—a recurrent functional theme across CRC-associated bins examined further below—was encoded in a low-monophyly bin (bin 200, monophyly = 0.23), consistent with a cancer-associated “side hustler” mechanism potentially shared across *Fusobacterium* sub-clades.

### Temporal stability of gene bins within individual gut microbiomes

**Figure 5.**
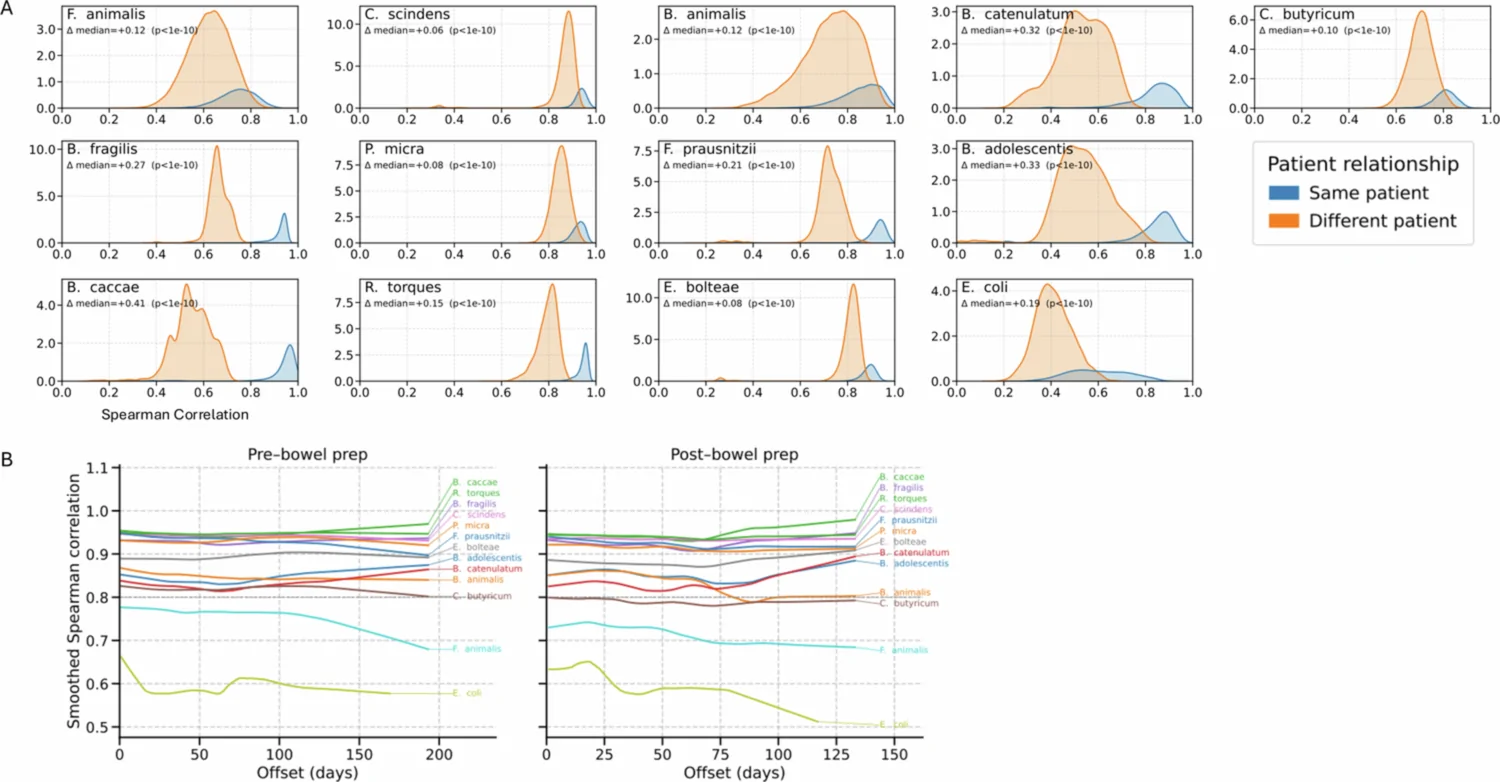
Temporal autocorrelation of bin abundances. **a,** Kernel density estimate (KDE) plots comparing Spearman correlations between bin abundances across longitudinal sampling timepoints from the Fukuyama dataset. Correlations are stratified by patient relationship and study interval. **b**, Changes in Spearman correlations of bin abundances as a function of increasing sample intervals, shown separately for each bacterial species.

A practical question in translating findings from CRC microbiome studies to designing a microbiome-based screening test is whether the key microbial signals are stable over time. Thus, we next sought to characterize the temporal stability of our pangenome bin-based framework and to define the timescales over which our framework might be applied. Robustness of our analyzes was evaluated using longitudinal samples from the Fukuyama cohort,^19^ performing autocorrelation calculations of bin densities within and between individuals. Across all bacterial species, intra-individual bin abundance correlations were higher than correlations between individuals. (*Δ* median intra- minus inter-individual ranged from +0.06 to +0.41, *p* < 10^−10^) (Figure 5A).

For most of the species we examined, bin-level structure also remained stable before versus after colon cleanout, suggesting that even large acute perturbations do not induce substantial shifts in microbial gene bin abundances (*Δ* median pre- minus post-bowel prep ranged from −0.00 to +0.04, *p* < 0.04) (Figure 5B and **S5**). Moreover, at differing time intervals, pangenome bin density correlations were overwhelmingly invariant, supporting the assumption that the pangenome bin structure is temporally persistent (Figure 5B). Notably, two bacterial species, *Escherichia coli* and *Fusobacterium animalis*, displayed comparatively lower within-individual time-dependent correlations, suggesting a more tenuous niche in the gut microbial ecosystem and hence greater potential temporal variability in their pangenome profiles relative to other taxa.

### Synergistic interspecies gene bin associations in CRC

**Figure 6.**
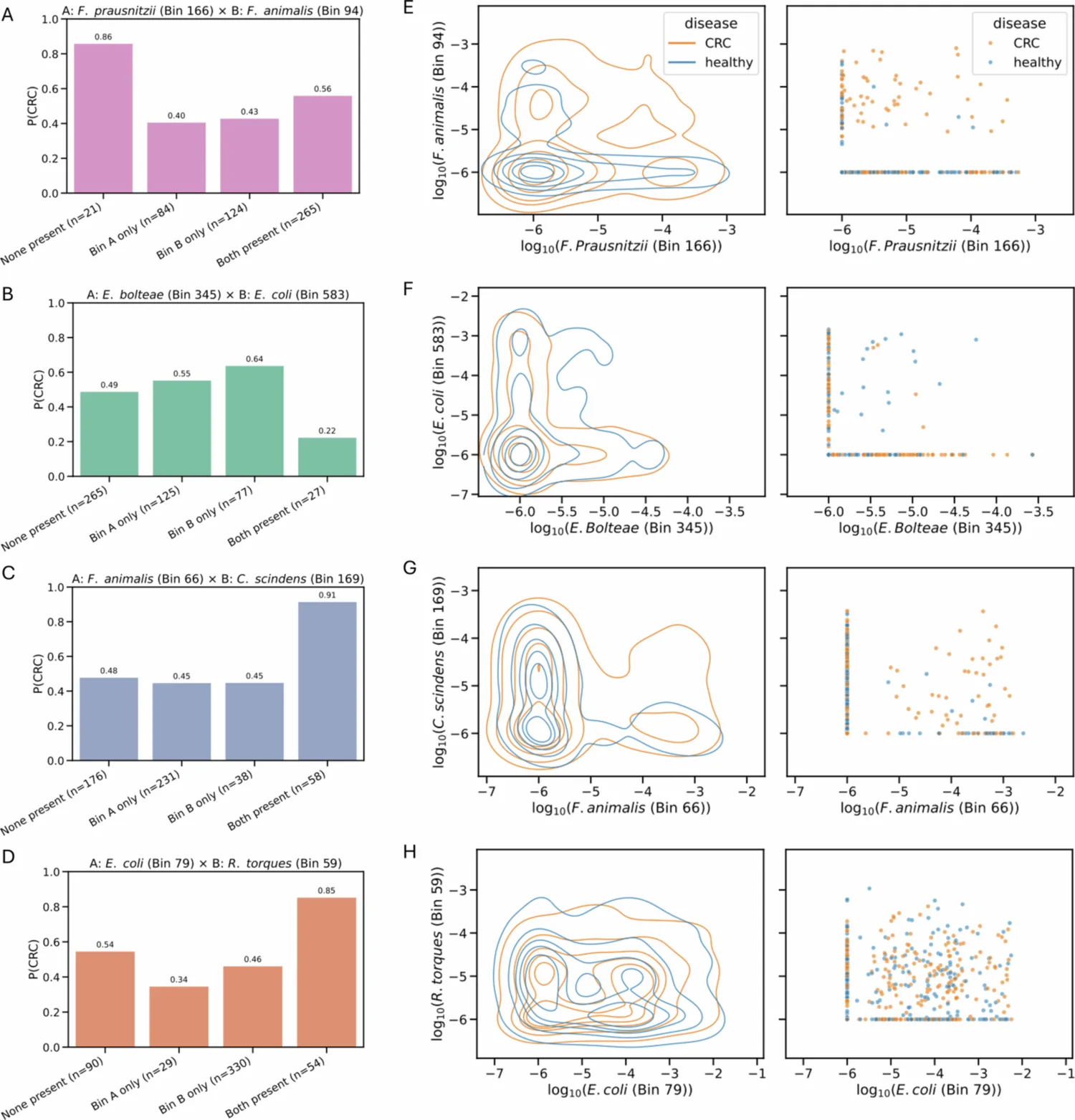
Synergistic and antagonistic CRC/health-associated bins. **a-d**, Logistic regression-derived probabilities of CRC conditional on the presence or absence of specific pangenome bins contained within the Yachida dataset. **e-h,** KDE and scatter plots showing the bin abundance distributions of synergistic and antagonistic interactions corresponding to the combinations evaluated in a-d.

Envisioning that ecological and evolutionary relationships between bacteria in CRC microbiomes are embedded in our gene bin analysis, we next asked whether gene bins from different species might act together, either reinforcing or opposing each other's association with CRC. To test this, we evaluated all pairwise bin combinations using binary logistic regression models incorporating interaction terms. Models were ranked by nominal interaction-term significance, and the top 200 associations were retained for exploratory evaluation (**Table S10**). Given the high-dimensional search space, these results were used to prioritize candidate non-additive associations for biological interpretation rather than to establish direct interspecies interactions. Among the highlighted interactions, six of the eight pangenome bins had low monophyly scores (<0.14) and low prevalence across pangenomes (**Table S3**), indicating that these interactions predominantly involve “side hustler” elements—phylogenetically dispersed, low-prevalence gene clusters—consistent with our broader observation that the CRC microbiome is shaped by mobile functional elements that do not act independently. We highlight four high-ranking candidate interactions (Figure 6).

Bin 166 of *F. prausnitzii* synergized with bin 94 of *F. animalis*, increasing CRC probability beyond either bin alone (P(CRC) = 0.56 vs. 0.40 and 0.43 respectively; *p* < 10⁻^5^, Figure 6A,E). This interaction is notable because *F. prausnitzii* is canonically health-protective, yet bin 166—a low-prevalence (3%), low-monophyly (0.13) “side hustler” element—inverts this protective association in the context of *F. animalis* co-presence. We speculate that bin 166 may encode horizontally acquired machinery that modifies the local tumor microenvironment, for instance through metabolite production, oxygen depletion, or immune modulation, in ways that amplify *F. animalis* fitness or its known oncogenic activities.

Bin 345 from *E. bolteae* demonstrated a unique antagonistic interaction with bin 583 from *E. coli*, wherein co-presence was linked to markedly reduced CRC probability relative to either bin alone (P(CRC) = 0.22 vs. 0.49 and 0.55; *p* < 0.001, Figure 6B and F). Both bins are low-monophyly “side hustler” elements present in only ~9-12% of strains, and neither shows individual CRC association approaching statistical significance, making their joint protective effect particularly striking. One possibility is that co-carriage of these bins marks a specific host microenvironment, perhaps one with robust mucosal immunity or favorable dietary substrate availability, that is intrinsically protective against CRC. Alternatively, their co-presence may reflect competitive metabolic interactions that displace genuinely oncogenic bacteria or alter bile acid and short-chain fatty acid profiles in ways that are protective.

Bin 66 from *F. animalis* synergized with bin 169 from *C. scindens* to produce the strongest CRC association observed in this analysis (P(CRC) = 0.91 with co-presence; *p* < 10⁻^5^, Figure 6C and G) relative to co-absence (0.48) or single-bin presence (~0.45). *C. scindens* is among the gut bacteria most capable of converting primary to secondary bile acids via 7α-dehydroxylation, generating deoxycholic acid (DCA)^20^—a known tumor promoter that induces DNA damage and suppresses colonocyte apoptosis. We hypothesize that if bin 169 encodes or upregulates bile acid modification capacity, and bin 66 in *F. animalis* encodes complementary functions such as bile acid transport or resistance, their co-presence could establish a synergistic oncogenic niche in which *C. scindens* generates DCA while *F. animalis* strains carrying bin 66 thrive in that bile acid-enriched environment and amplify their own CRC-associated activities.

Bin 79 from *E. coli* and bin 59 from *R. torques* showed a pattern in which co-presence of both bins was strongly associated with CRC (P(CRC) = 0.85), while presence of either bin alone was associated with substantially lower CRC probability (0.34 and 0.46 respectively; *p* < 0.001, Figure 6D and H). Notably, *R. torques* bin 59 has a monophyly score of 1.0—a perfect specialist, ancestrally conserved across all reference genomes that carry it—yet is present in only 7% of strains, making it rare but highly lineage-restricted. Its co-occurrence with *E. coli* bin 79 producing a strong synergistic CRC signal suggests that this combination marks a specific oncogenic ecological state. We speculate that *R. torques* bin 59, given its perfect monophyly, may encode a conserved capacity—perhaps related to mucin degradation or polysaccharide utilization consistent with *R. torques*'s known role as a *Lachnospiraceae* ^21^—that creates a niche permissive to the CRC-associated activity encoded by *E. coli* bin 79. The high monophyly of bin 59 makes it an attractive candidate for biomarker development: its lineage-conserved nature implies cross-cohort reliability, and its synergistic interaction with a low-monophyly side-hustler element illustrates how specialist and side-hustler gene content can interact to produce emergent oncogenic risk beyond what either contributes alone.

Taken together, these interactions underscore the value of a pangenome bin-based approach in revealing higher-order relationships that are invisible to species-level analysis, and support a view of the gut microbiome as an ecosystem of interacting genetic elements. The predominance of side-hustler elements among the interacting bins is consistent with the broader thesis that mobile, horizontally acquired gene content, rather than fixed species-level composition, shapes the functional ecology of the CRC microbiome.

### CRC-associated pangenome bins share annotated gene functions

**Figure 7.**
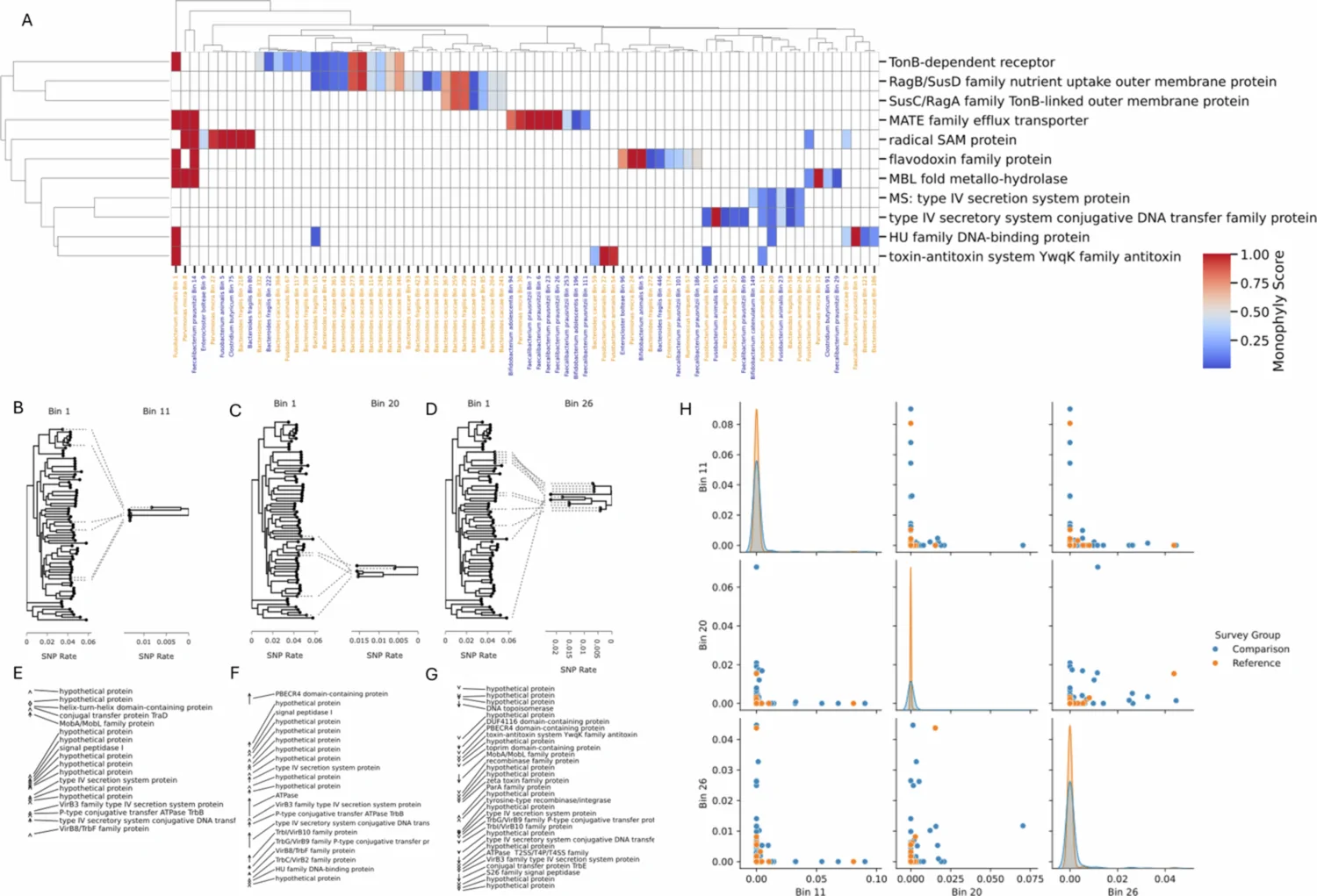
Shared annotated gene functions across bins. **a,** Identification of proteins shared across multiple bins and bacterial species. Bins are clustered according to monophyly scores in a gene presence heatmap. **b-d,** Phylogenetic comparison between bins of interest with low monophyly scores and containing Type IV secretion system (T4SS) genes and the reference genome. **e-g**, Gene contents of selected bins. **h,** Bin abundance scatterplots for all combinations of selected bins, stratified by sample status as comparison (CRC) or reference (control).

Given that CRC- and health-associated pangenome bins are taxonomically dispersed and often non-monophyletic, we next evaluated whether these gene bins shared common gene functions. We systematically examined the functional annotations of all pangenome bins for every bacterial species and identified proteins recurring across multiple CRC and health-associated pangenome bins. This analysis identified fourteen protein families present in at least five distinct gene bins across multiple bacterial species (Figure 7A). Notably, proteins detected in our univariate analysis—including TonB-dependent receptor, Type IV secretion system, and RagB/SusD—were observed among these shared functional categories. Thus, despite broad phylogenetic spread, CRC-associated pangenome bins show functional convergence across multiple taxa. *Fusobacterium animalis* bins 11, 20, and 26 each encode Type IV secretion system proteins, representing gene clusters with variable phylogenetic origins yet shared functionality (Figure 7B–G). Analysis of bin abundances substantiates this observation, revealing univariate associations with CRC and health, but little evidence for coordinated associations among bins encoding similar functionalities (Figure 7H).

## Discussion

Taxonomic and clade-based frameworks remain the prevailing standard for studying the microbiome, including in studies identifying bacteria enriched in healthy or colorectal cancer (CRC) states.^9^ ^,^ ^17^ Here we explored the use of pangenome bins as an alternative lens through which to characterize microbial gut environments in CRC, with the goal of enhancing functional gene-level resolution of microbial CRC correlates while explicitly accounting for non-cladistic evolutionary processes such as horizontal gene transfer (HGT). We quantified the phylogenetic coherence of each pangenome bin relative to the core-genome phylogeny through a monophyly score, where high values indicate complete conservation within a subspecies lineage (Figure 2A). Using this framework, we identified pangenome bins (typically corresponding to operons) across bacterial species which were significantly associated with either CRC or health. Examination of their phylogenetic distributions revealed substantial variability in monophyly both within and across species, consistent with a mixture of vertically inherited traits and genes subject to frequent gain or loss, potentially via HGT.

When comparing monophyly score distributions of CRC- or health-associated bins to unassociated bins, only two significant differences emerged from 26 comparisons, both within *F. prausnitzii*. The species is generally regarded as a health-associated bacteria and a major butyrate producer.^22-24^ However, we previously found this relationship to be more complex, with identification of significant CRC and health-linked genomic features within this species.^3^ Our findings here now offer a potential explanation for this apparent paradox: health-linked gene bins exhibited higher monophyly scores, consistent with lineage-conserved traits, whereas CRC-associated bins displayed lower monophyly scores, consistent with more polyphyletically dispersed (possibly mobile) gene bins. In other words, the health-associated functions may contribute to stable niche adaptation and are therefore maintained across particular *F. prausnitzii* lineages. In contrast, the lower phylogenetic coherence of CRC-associated bins suggests more evolutionarily labile gene content whose selective fitness is highly dependent on environmental context.

The absence of similar findings in the remaining bacteria may reflect limited power or more heterogeneous genomic architectures. The latter case would suggest that contributions to CRC risk or protection are unlikely to be attributable to a single species or stable subspecies. Instead, this points toward a model where variations in genetic content within bacterial species, rather than each species as a whole, are the drivers of selection in the CRC-associated gut environment.

Beyond univariate associations, we also examined whether combinations of pangenome bins exhibited synergistic or antagonistic relationships with CRC. This exploratory analysis identified several candidate non-additive associations, highlighting examples in which the joint presence or absence of specific bins was associated with CRC risk in ways not predictable from individual effects alone. These findings underscore the value of a pangenome bin-based approach in revealing higher-order relationships and support a view of the gut microbiome as an ecosystem of interacting genetic elements, rather than a collection of discrete, independently acting species-level contributors. Our results inform testable hypotheses and motivate follow-up experiments. For example, for the 4 examples illustrated in Figure 6: (i) co-culturing *F. prausnitzii* strains stratified by bin 166 genotype with *F. animalis* in colonoid or murine colonization models, and then measuring *F. animalis* abundance and markers of epithelial invasion, would directly test the hypothesis that bin 166 from *F. prausnitzii* promotes colonization efficiency, fitness, and/or oncogenicity. (ii) Given *E. bolteae*'s known capacity for bile acid metabolism^25^ and *E. coli*'s competitive interactions with more virulent strains, future work characterizing the metabolic output of strains carrying these bins in co-culture—specifically measuring secondary bile acid profiles and competitive exclusion of *F. animalis*—would test whether the protective signal reflects a direct functional interaction or a shared ecological context. (iii) The hypotheses that either *C. scindens*-generated DCA promotes *F. animalis* oncogenicity or that *F. animalis* enables *C. scindens* to more robustly generate DCA could be tested by measuring 7α-dehydroxylase activity and DCA production in *C. scindens* strains stratified by bin 169 presence, and by testing whether *F. animalis* strains carrying bin 66 show differential growth or T4SS activation in DCA-supplemented *in vitro* models. (iv) Synergy of bin 79 from *E. coli* and bin 59 from *R. torques* could be directly tested in *in vitro* co-culture experiments involving bacterial strains possessing or lacking these genes.

Although these interaction patterns generate biologically plausible hypotheses, they should not be interpreted as evidence of direct interspecies interactions. The observed synergism may instead reflect shared adaptation to features of the CRC-associated gut environment, with the two organisms responding to common ecological pressures to different extents. Such pressures could include altered nutrient availability, dependence on a third microbial species, or host immune activity. Experimental validation in controlled co-culture systems and animal models will therefore be necessary to distinguish direct functional interactions from indirect ecological associations.

Building on these observations, we next investigated the functional composition of CRC- and health-associated gene bins to determine whether shared biological themes emerged across bins and across species. This analysis identified several proteins of potential relevance that recurred across multiple associated bins. Prominent examples include components of Type IV secretion systems, which have been implicated in the dissemination of mobile genetic elements and antibiotic resistance;^26-29^ TonB-dependent receptors, which mediate iron-siderophore transport in Gram-negative bacteria and may contribute to iron availability,^30^ a factor associated with increased bacterial fitness and poorer CRC prognosis;^31^ RagB/SusD family nutrient uptake proteins, which facilitate the active import of complex nutrients and have been linked to host-microbe interactions, and whose selective value depends on the availability of complex dietary polysaccharides in the gut lumen;^32-35^ and flavodoxins, which play roles in oxidative stress resistance and virulence in organisms such as *Candida albicans* and are important for survival in *Helicobacter pylori*.^36^ ^,^ ^37^ The recurrence of these functions across phylogenetically distinct CRC-associated bins, often with low monophyly scores, therefore suggests convergence on functions related to genetic exchange, nutrient acquisition, and persistence rather than dependence on a single bacterial lineage.

An important implication of these findings is that the evolutionary mechanisms shaping CRC-associated microbial functions may differ substantially across traits and species. Polyphyletic distributions are suggestive of repeated horizontal acquisition or loss of genetic elements, raising the possibility that adaptive functions can disseminate independently of species boundaries. Such mobility could accelerate microbial adaptation to the tumor-associated niche by allowing diverse lineages to converge on similar functional capabilities without requiring the expansion of a single successful clone. However, low monophyly alone does not constitute direct evidence of horizontal gene transfer. Alternative evolutionary processes, such as ancient gene polymorphisms, may also contribute to discordance between gene bin and species phylogenies. Future analyzes integrating genomic context and synteny will be imperative for distinguishing these mechanisms and more definitively determining the extent to which horizontal gene transfer shapes oncogenic microbial ecology.

Several limitations warrant consideration. First, our analysis was restricted to 13 bacterial taxa. Although CAG associations were evaluated across the Yachida, Hannigan, and Zeller cohorts, additional taxa and populations will be needed to establish the broader generalizability of this framework. Archaea, fungi, and viruses were outside the scope of the present bacterial pangenome analysis and may contribute additional CRC-associated functions. Second, we do not perform community-level analyzes of alpha and beta diversity. While we have the ability to estimate the proportion of genome copies from each species which contain a particular operon, we could not estimate the total number of unique subspecies genotypes within each sample. Third, our analysis used reference-genome pangenomes and short-read mapping rather than de novo assembly of individual metagenomes. Nevertheless, incomplete strain representation in reference databases, reference-assembly errors, ambiguous mapping among homologous genes, and co-abundance-based assignment of genes to CAGs may affect bin definitions and abundance estimates. Fourth, functional inference was constrained by submitter-provided gene annotations. Although AlphaFold-assisted annotation improved functional predictions,^38^ a substantial fraction of bin-encoded proteins remain uncharacterized (**Tables S4-S9**). Finally, while monophyly scores provide a useful proxy for evolutionary distribution, they cannot definitively distinguish between ancient polymorphism and recent horizontal gene transfer without complementary analyzes of mobile genetic elements or genomic context.

Nevertheless, we observe overall trends in the phylogenetic coherence of CRC- and health-associated genetic elements which provide important insight into the evolutionary dynamics of the microbiome-CRC connection. We had originally hypothesized that CRC-associated pangenome bins would have lower monophyly scores (where reproductive success in that environment was a “side-hustle” and not the committed evolutionary strategy of a particular subspecies lineage). However, we observed a high degree of variation in evolutionary trends both within and between species. For example, *F. animalis* contains numerous CRC-associated pangenome bins spanning high and low monophyly scores (Figures 2F, 7B–D). This pattern suggests that its accessory genome contains functionally related elements with distinct evolutionary histories: some gene clusters may have become conserved within particular lineages, whereas others remain evolutionarily labile and undergo repeated gain or loss. Thus, selection within the CRC-associated gut environment may act on functional capacity through both conserved and flexible components of the *F. animalis* accessory genome. In contrast, all of the CRC-associated bins within *P. micra* have high monophyly scores, suggesting the existence of one or more reproductively isolated lineages with higher growth rates in the context of CRC. Conversely, the CRC-associated bins of *F. prausnitzii* all have low monophyly scores, suggesting that those genes with a selective advantage in CRC are exchanged between lineages frequently–anthropomorphically referred to as a “side hustle.” Overall, we observe a diversity of evolutionary patterns both for CRC- and health-associated genetic elements. This suggests that any cellular function with a proposed connection to CRC (e.g. Type IV secretion, iron uptake, and RagB/SusD nutrient acquisition) may be subject to horizontal gene transfer in the natural environment, depending on the organism, environmental condition, or any number of other factors. We strongly recommend that genomic plasticity and evolutionary selection be considered in further host-microbiome studies for CRC.

Future work should extend this framework to additional taxa and cohorts and incorporate plasmid association and synteny to more directly evaluate HGT. Experimental studies will also be needed to determine whether the identified pangenome bins contribute to CRC development or instead reflect adaptation to the tumor-associated niche. From a translational perspective, lineage-conserved CAGs may support strain-specific biomarkers, whereas shared functional categories may provide signals that are more robust to taxonomic variation across populations. Their diagnostic value should be compared directly with species-level models in prospective cohorts, and therapeutic development will require evidence that candidate functions—such as secretion systems or nutrient-acquisition pathways—causally contribute to disease and can be targeted without disrupting beneficial microbial activity. Together, these findings support further evaluation of function-centric approaches to microbiome-based CRC diagnosis and intervention.

## Materials and methods

### Constructing pangenome reference databases

The pangenome reference database for each bacterial species was generated using the gig-map/build_pangenome.nf Nextflow workflow (https://github.com/FredHutch/gig-map/build_pangenome.nf) (commit 35dd28389c).

Analysis steps include:

1. Deduplicating protein-coding gene content across all genomes (CD-HIT);
2. Identifying genome membership for each unique gene reference (DIAMOND);
3. Unsupervised clustering of genes into pangenome bins (scipy).

For each species, a list of available reference genomes was obtained from the NCBI Genome database (https://www.ncbi.nlm.nih.gov/datasets/genome/). Filtered genomes to attain approximately 100 high quality genomes. Atypical genomes and MAGs were excluded and genomes with NCBI RefSeq annotations were prioritized. Assembly level was adjusted to the highest level feasible with consideration of sample size. That table of genomes was provided to the build_pangenome.nf workflow using the genome_tsv parameter. Other parameters provided to the workflow were:

• cluster_coverage: 0.9
• cluster_similarity: 0.9
• min_identity: 90
• max_overlap: 50
• min_genomes_per_gene: 2
• max_dist_genes: 0.05
• ftp_threads: 1
• aligner: diamond
• max_dist_genomes: 0.05
• min_bin_size: 1
• max_evalue: 0.001
• query_gencode: 11
• min_gene_length: 50
• min_coverage: 90

### Estimating relative abundance of pangenome bins within metagenome datasets

Each metagenome dataset was analyzed against each pangenome reference database using the gig-map/align_pangenome.nf Nextflow workflow (https://github.com/FredHutch/align_pangenome.nf) (commit 35dd28389c).

Analysis steps include:

1. Aligning each metagenomic read against the deduplicated gene catalog (DIAMOND);
2. Resolving any reads which align to multiple genes with equally high quality (FAMLI);
3. Calculating relative abundance in terms of RPKM (reads per kilobase, per million reads aligned).

The collection of FASTQ files available for each metagenome dataset were captured as a CSV with the columns “sample”, “R1”, and “R2”, which was provided to the align_pangenome.nf workflow using the “samplesheet” parameter. The annotations for each sample in the dataset (e.g. CRC vs. control) were captured as a CSV and provided to the workflow using the “metadata” parameter. The parameters “genes”, “group_profile”, “gene_bins”, and “genome_groups” were used to map inputs from the pangenome reference database. Additional parameters provided to the workflow were:

‐ max_evalue: 0.001
‐ min_identity: 90
‐ min_n_reads: 1
‐ min_score_reads: 50
‐ min_n_genes: 1

The Cirro Data Platform (https://cirro.bio) was used to manage the execution and management of the pangenome build and metagenome alignment steps, keeping track of the software versions used for each step of the analysis and the provenance of all analysis results.

### Identifying pangenome bins with significantly different relative abundances in CRC vs. healthy samples

The relative abundance (RPKM) of each pangenome bin was compared between the CRC and control groups using the Kruskal-Wallis test. For a two-group comparison the Kruskal-Wallis test is equivalent to the Mann-Whitney U test, and results are reported as the Mann-Whitney U test throughout the Results and figure legends. False Discovery Rate (FDR) correction was applied using the Benjamini-Hochberg (BH) procedure (implemented by the statsmodels.stats.multitest module in Python) to calculate q-values. A q-value of ≤0.2 was considered significant. Prior to calculating q-values, data was filtered to exclude gene bins containing less than five genes.

The predefined CAGs were quantified in the independent Hannigan and Zeller cohorts without reclustering. Concordance was assessed among the 3,505 CAGs quantified in all three cohorts. To generate the null distribution, CRC association status—positive, negative, or nonsignificant—was independently permuted among CAGs within each cohort while preserving the observed number of CAGs in each association category. The observed numbers of positively associated CAGs shared across exactly two or all three cohorts were compared with the corresponding permutation distributions.

### Calculating monophyly score for pangenome bins

Monophyly is the degree to which the distribution of a trait corresponds to a monophyletic grouping within a larger population. The monophyly score was calculated using the ANI-based whole-genome phylogeny. For each pangenome bin, the monophyly score was calculated as the ratio of the number of genomes in which that group of genes was observed, divided by the smallest possible monophyletic grouping of genomes which also included all of those genomes. Values for the score range from 0 to 1.

### Pairwise interaction analysis

All pairwise combinations of pangenome bins were evaluated in the Yachida cohort using binary logistic regression models of the form CRC status ~ bin A + bin B + (bin A x bin B). Bin presence was defined as abundance > 0. A total of 980,001 models were fit. Models were ranked by the nominal *p*-value of the interaction term, and the 200 most significant were retained (**Table S10**).

### Temporal autocorrelation of pangenome bin abundances

Longitudinal samples from the Fukuyama cohort were used to assess the temporal stability of bin abundances. For each bacterial species, Spearman correlations were computed between bin abundance vectors from pairs of samples, stratified by whether the two samples originated from the same or different individuals and by the time interval separating them. Intra- and inter-individual correlation distributions were compared using two-sided Mann-Whitney U test.

### Functional annotation of genes in pangenome bins

Genes from the pangenome bins were annotated using NCBI RefSeq annotations. In addition, the subset of genes in pangenome bins listed in **Tables S4-S9** were annotated with the AlphaFold Protein Structure Database (https://alphafold.ebi.ac.uk/) using default parameters.

## Supplementary Material

Tables_Supplementary_260731.xlsx Tables_Supplementary_260731.xlsx

Supplementary Material SUPPLEMENTAL TABLE AND FIGURE CAPTIONS

Figures_Supplementary.pdf

## Data Availability

Shotgun metagenomic sequencing data were downloaded from the NCBI Sequence Read Archive (SRA) repository under NCBI BioProject IDs PRJDB4176, PRJNA389927, PRJEB6070, and PRJNA388263; and from the European Nucleotide Archive under accession ERP005534.
