# Supplementary material for: Pangenome analysis reveals both niche-specific “specialists” and microbial “side hustlers” in colorectal cancer microbiomes
Source: Gut Microbes. 2026 Sep 15;18(1):2728331. doi: 10.1080/19490976.2026.2728331 (PMC13580483; doi:10.1080/19490976.2026.2728331)
Supplement: Supplementary file 1 [file KGMI_A_2728331_SM7178.pdf]

Figure S1

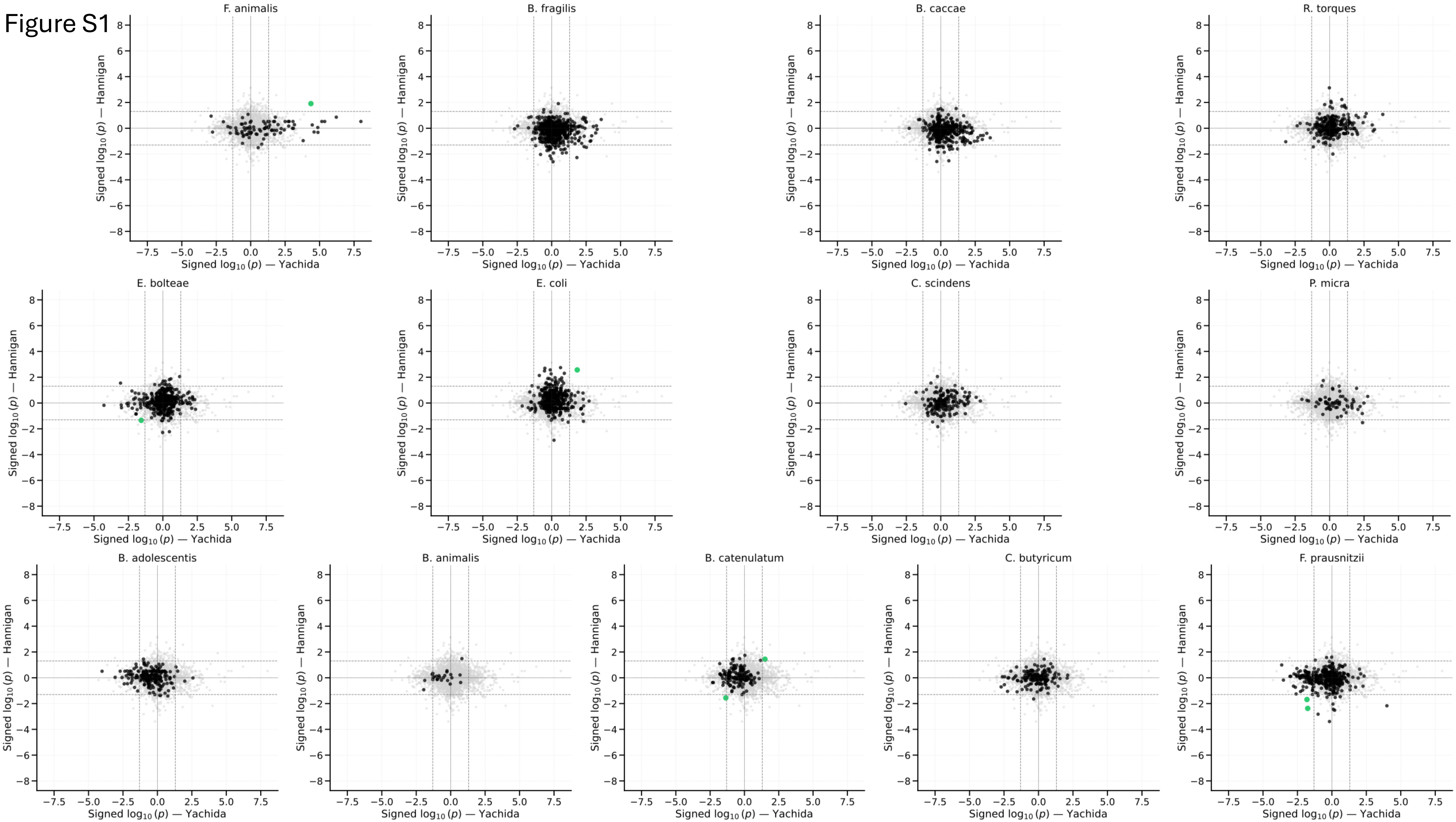
F. animalis
B. fragilis
B. caccae
R. torques
E. bolteae
E. coli
C. scindens
P. micra
B. adolescentis
B. animalis
B. catenulatum
C. butyricum
F. prausnitzii
Signed log10 (p) — Hannigan
Signed log10 (p) — Yachida

Figure S2

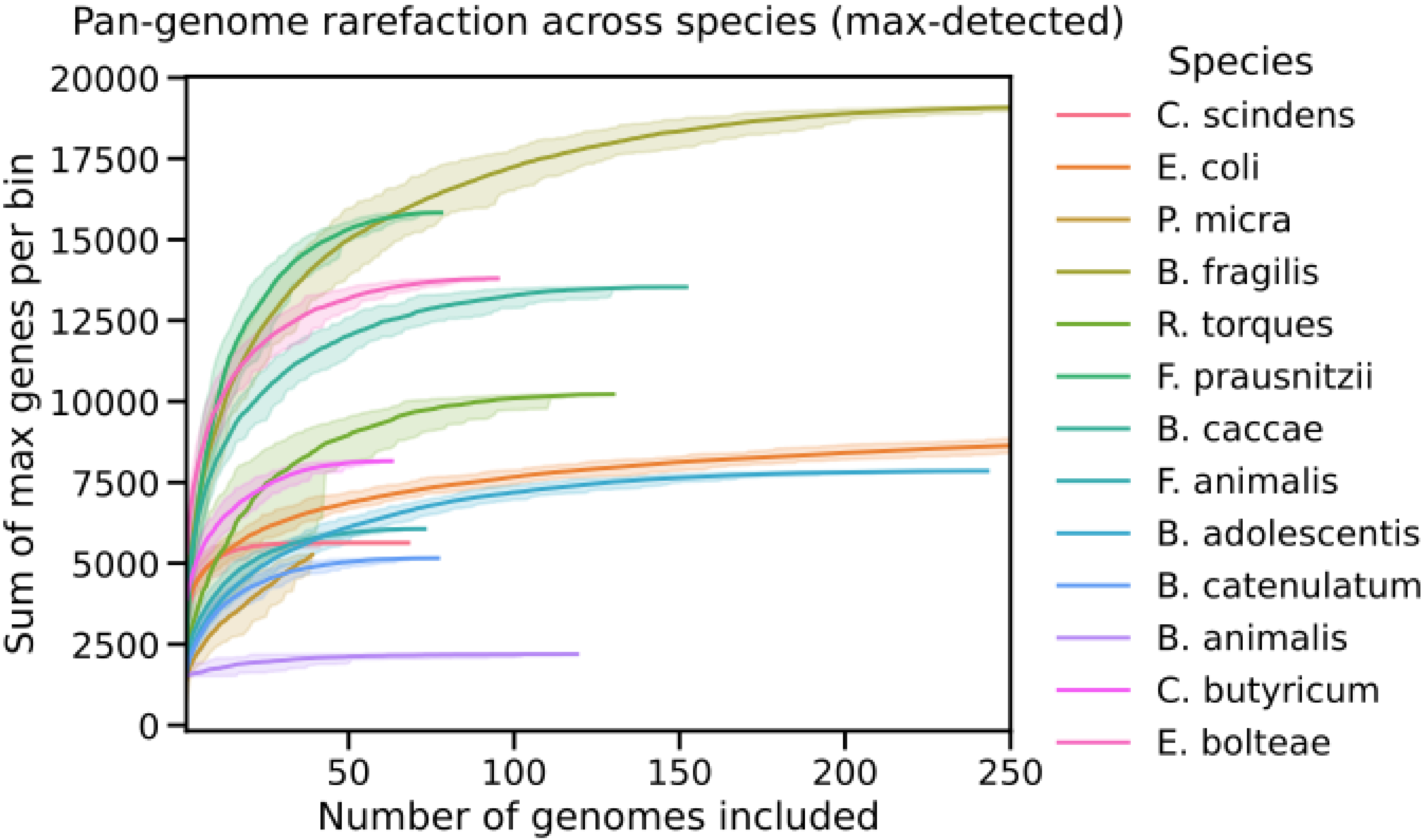

Pan-genome rarefaction across species (max-detected)
Sum of max genes per bin
20000
17500
15000
12500
10000
7500
5000
2500
0
50
100
150
200
250
Number of genomes included
Species
C. scindens
E. coli
P. micra
B. fragilis
R. torques
F. prausnitzii
B. caccae
F. animalis
B. adolescentis
B. catenulatum
B. animalis
C. butyricum
E. bolteae

Figure S3

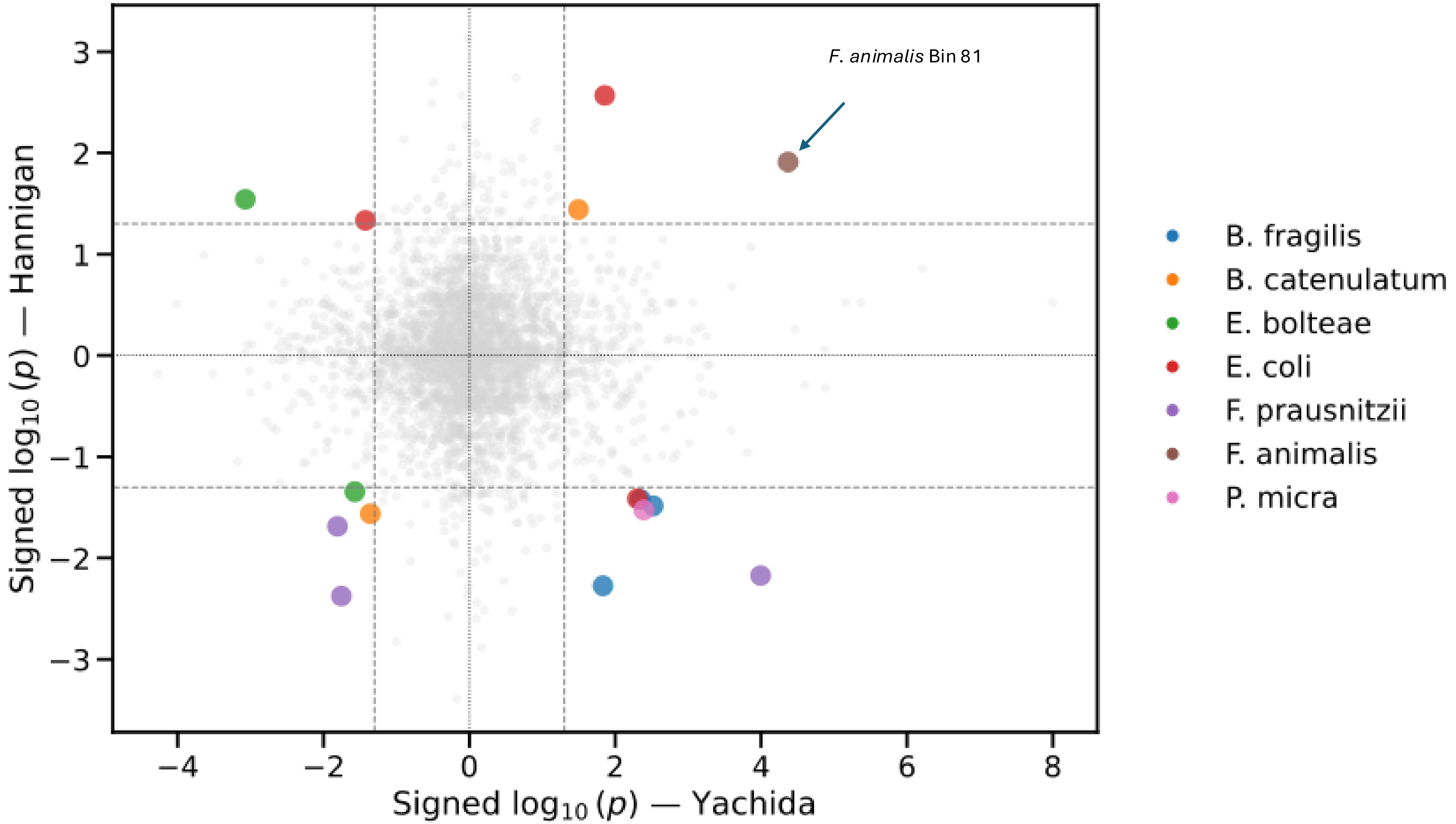
F. animalis Bin 81
Signed log10 (p) — Hannigan
Signed log10 (p) — Yachida
3
2
1
0
−1
−2
−3
−4
−2
0
2
4
6
8
B. fragilis
B. catenulatum
E. bolteae
E. coli
F. prausnitzii
F. animalis
P. micra

Figure S4

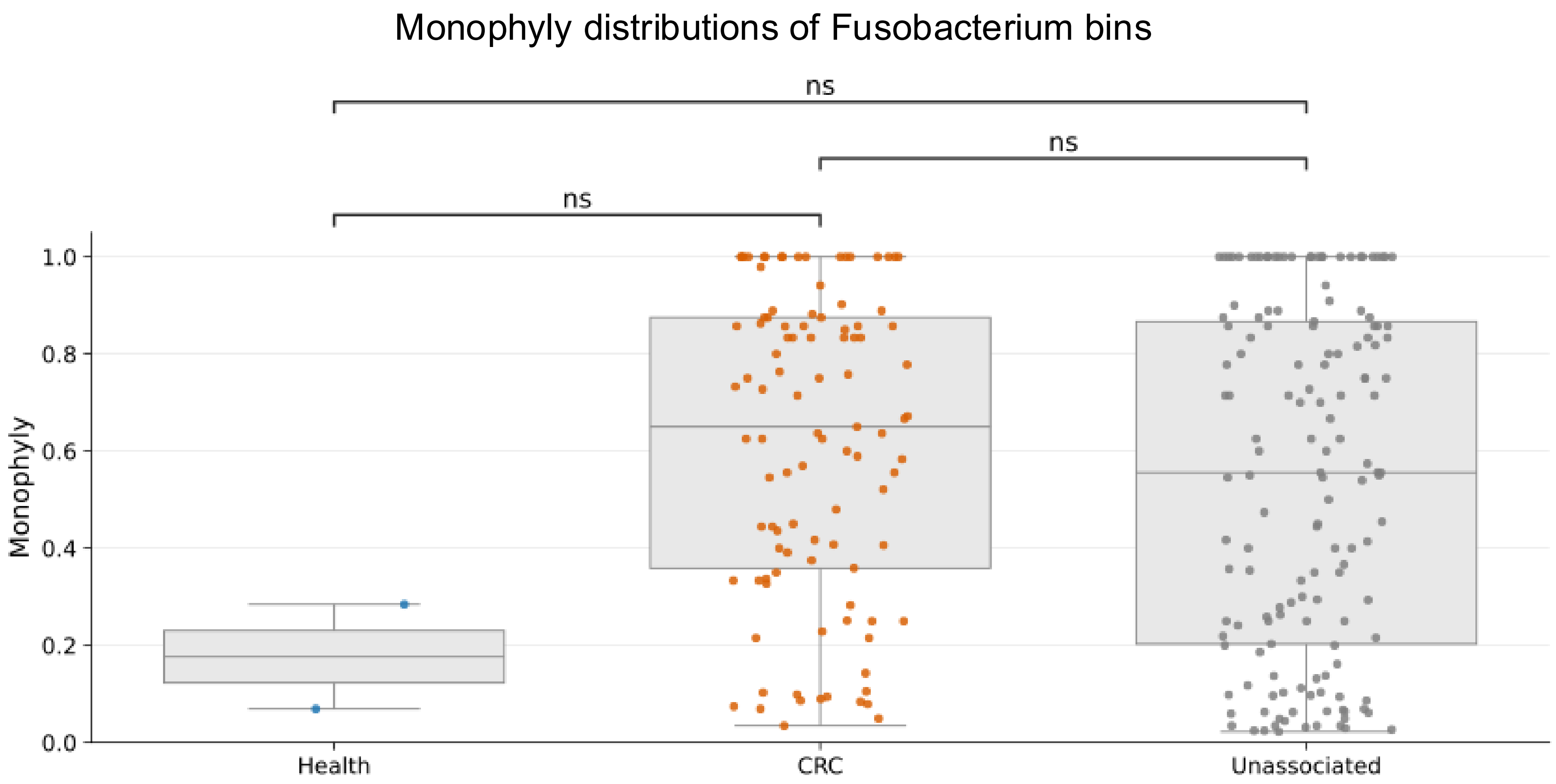
Monophyly distributions of Fusobacterium bins
ns
ns
ns
Monophyly
1.0
0.8
0.6
0.4
0.2
0.0
Health
CRC
Unassociated

Figure S5

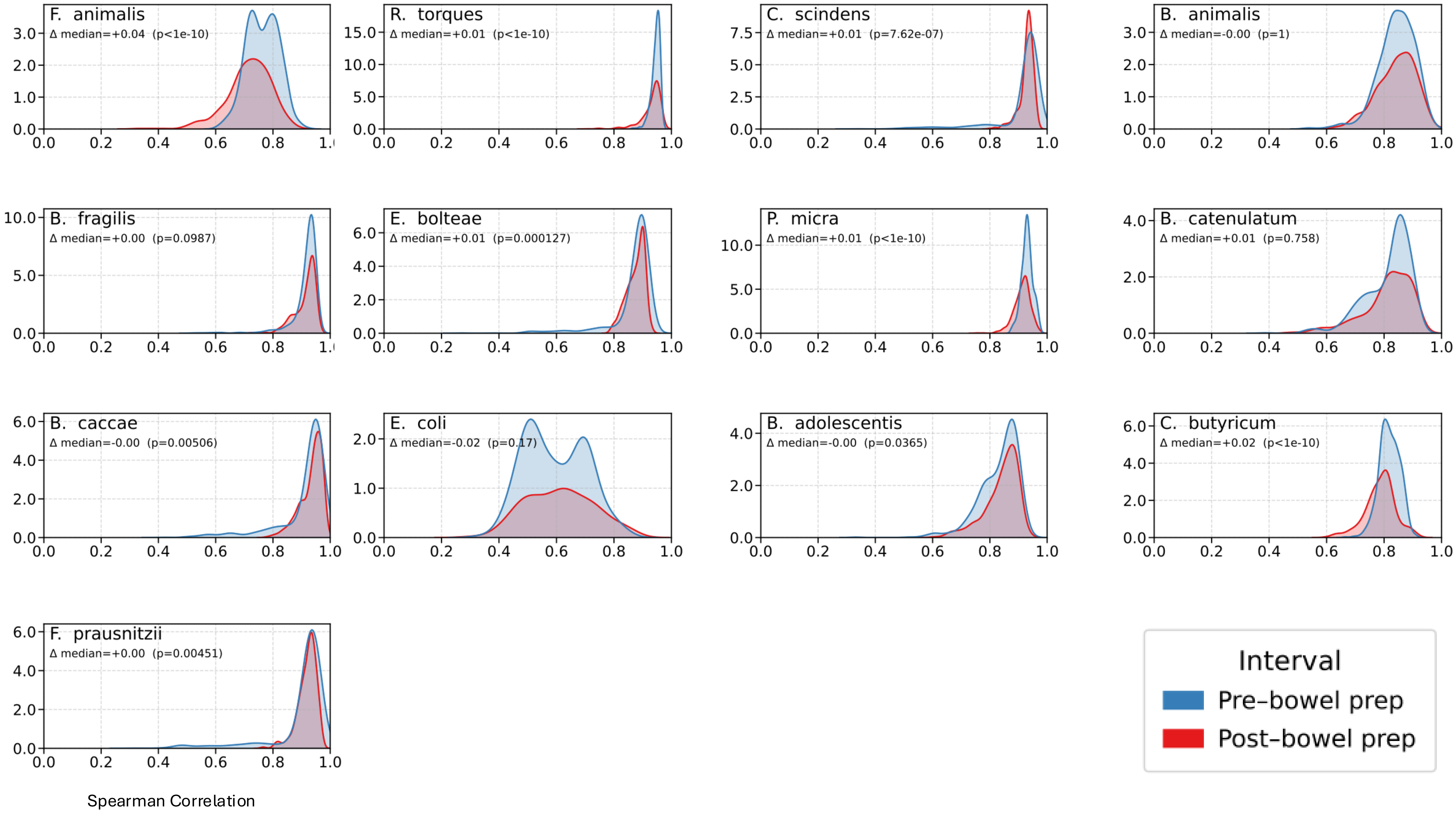
F. animalis
Δ median=+0.04 (p<1e-10)
R. torques
Δ median=+0.01 (p<1e-10)
C. scindens
Δ median=+0.01 (p=7.62e-07)
B. animalis
Δ median=-0.00 (p=1)
B. fragilis
Δ median=+0.00 (p=0.0987)
E. bolteae
Δ median=+0.01 (p=0.000127)
P. micra
Δ median=+0.01 (p<1e-10)
B. catenulatum
Δ median=+0.01 (p=0.758)
B. caccae
Δ median=-0.00 (p=0.00506)
E. coli
Δ median=-0.02 (p=0.17)
B. adolescentis
Δ median=-0.00 (p=0.0365)
C. butyricum
Δ median=+0.02 (p<1e-10)
F. prausnitzii
Δ median=+0.00 (p=0.00451)
Spearman Correlation
Interval
Pre–bowel prep
Post–bowel prep
