# Supplementary material for: Pangenome analysis reveals both niche-specific “specialists” and microbial “side hustlers” in colorectal cancer microbiomes
Source: Gut Microbes. 2026 Sep 15;18(1):2728331. doi: 10.1080/19490976.2026.2728331 (PMC13580483; doi:10.1080/19490976.2026.2728331)
Supplement: Supplementary file 2 [file KGMI_A_2728331_SM1014.docx]

**SUPPLEMENTAL TABLE AND FIGURE CAPTIONS**

**Table S1.** Bacterial taxa analyzed in this paper.

**Table S2A.** Significant bins (p-values) in Yachida, Hannigan, and Zeller datasets.

**Table S2B.** Significant bins (FDR-corrected p-values) in Yachida dataset.

**Table S2C.** Observed cross-study concordance compared to null permutation.

**Table S2D.** Concordant species and gene bins between Yachida, Hannigan, and Zeller datasets.

**Table S3.** Monophyly and prevalence values for bins selected shown in the Figure 6 interdependency analysis.

**Table S4.** *Fusobacterium*, bin 58 annotated contents.

**Table S5.** *Fusobacterium*, bin 37 annotated contents.

**Table S6.** *Fusobacterium*, bin 200 annotated contents

**Table S7.** *Fusobacterium animalis*, bin 11 annotated contents

**Table S8.** *Bacteroides fragilis*, bin 15 annotated contents

**Table S9.** *Bacteroides caccae*, bin 85 annotated contents

**Table S10.** Interdependency logistic regression results (200 most significant)

**Figure S1.** Scatterplot comparison of signed log_10_(p) for each bin shared between the Yachida and Hannigan datasets.

**Figure S2.** Rarefaction for each of the 13 bacterial species analyzed.

**Figure S3.** Scatterplot comparison of signed log_10_(p) with identification of concordant and discordant bins.

**Figure S4.** Monophyly distribution of *Fusobacterium* bins

**Figure S5.** Spearman correlation of each bacterial species before and after bowel prep.
